# Experimental transmission of enzootic nasal adenocarcinoma in sheep

**DOI:** 10.1186/1297-9716-44-66

**Published:** 2013-07-30

**Authors:** Scott R Walsh, Nicolle M Linnerth-Petrik, Darrick L Yu, Robert A Foster, Paula I Menzies, Andrés Diaz-Méndez, Heather J Chalmers, Sarah K Wootton

**Affiliations:** 1 Department of Pathobiology, Ontario Veterinary College, University of Guelph, Guelph, Ontario, Canada; 2 Department of Population Medicine, Ontario Veterinary College, University of Guelph, Guelph, Ontario, Canada; 3 Department of Clinical Studies, Ontario Veterinary College, University of Guelph, Guelph, Ontario, Canada

## Abstract

Enzootic nasal adenocarcinoma (ENA) is a contagious neoplasm of the secretory epithelial cells of the nasal mucosa of sheep and goats. It is associated with the betaretrovirus, enzootic nasal tumor virus (ENTV), but a causative relationship has yet to be demonstrated. In this study, 14-day-old lambs were experimentally infected via nebulization with cell-free tumor filtrates derived from naturally occurring cases of ENA. At 12 weeks post-infection (wpi), one of the five infected lambs developed clinical signs, including continuous nasal discharge and open mouth breathing, and was euthanized. Necropsy revealed the presence of a large bilateral tumor occupying the nasal cavity. At 45 wpi, when the study was terminated, none of the remaining infected sheep showed evidence of tumors either by computed tomography or post-mortem examination. ENTV-1 proviral DNA was detected in the nose, lung, spleen, liver and kidney of the animal with experimentally induced ENA, however there was no evidence of viral protein expression in tissues other than the nose. Density gradient analysis of virus particles purified from the experimentally induced nasal tumor revealed a peak reverse transcriptase (RT) activity at a buoyant density of 1.22 g/mL which was higher than the 1.18 g/mL density of peak RT activity of virus purified from naturally induced ENA. While the 1.22 g/mL fraction contained primarily immature unprocessed virus particles, mature virus particles with a similar morphology to naturally occurring ENA could be identified by electron microscopy. Full-length sequence analysis of the ENTV-1 genome from the experimentally induced tumor revealed very few nucleotide changes relative to the original inoculum with only one conservative amino acid change. Taken together, these results demonstrate that ENTV-1 is associated with transmissible ENA in sheep and that under experimental conditions, lethal tumors are capable of developing in as little as 12 wpi demonstrating the acutely oncogenic nature of this ovine betaretrovirus.

## Introduction

Enzootic nasal adenocarcinoma (ENA) is a neoplasm of the secretory epithelial cells of the nose of sheep and goats [1]. ENA tumors can arise unilaterally or bilaterally, originating from the ethmoid turbinate and often expanding to occlude the nasal cavity. No metastasis has been reported in ENA cases, but disruption of the nasal septum structure as well as erosion of the cribiform plate has been reported [2,3]. Due to the cell type transformed and the space occupying nature of the tumor, the clinical signs of ENA include production of copious nasal exudate, open mouth breathing, dyspnea and facial asymmetry [1]. A neoplasm of the secretory epithelial cells of the distal lung of sheep, called ovine pulmonary adenocarcinoma (OPA), is known to be caused by jaagsiekte sheep retrovirus (JSRV) [4] and a similar etiology is suspected for ENA [5,6]. ENA is associated with the betaretrovirus, enzootic nasal tumor virus (ENTV), which is genetically very similar to JSRV [7]. ENTV is divided into two distinct sub species, one infecting sheep (ENTV-1) [7] and the other infecting goats (ENTV-2) [8]. Studies involving ENTV are hindered by the fact that there is no cell culture system for propagating the virus. Although a causal relationship between ENTV-1 infection and the development of ENA in sheep has not been proven, reverse transcriptase activity, ENTV-1 specific nucleotide fragments and antigens that cross react with antibodies against JSRV proteins are consistently found in nasal exudate as well as nasal tumor tissue [3,5,9,10]. ENA has been shown to be infectious in goats through intrasinus or intranasal inoculation of newborn goat kids with clarified nasal exudates pooled from three ENA affected goats [11]. In 1953, Cohrs reported transmission of ENA in sheep using cell and bacteria-free tumor filtrate [12], but more recently, similar experiments attempting transmission of ENA in sheep were unsuccessful (Dr James DeMartini, personal communication).

In this study, we tested the hypothesis that ENA can be induced in healthy l4-day-old lambs after exposure to nebulized cell-free tumor homogenate derived from sheep with ENA. We show that while the rate of tumor induction was low, clinical signs could be detected as early as 12 wpi. The clinical signs, histopathology, and tissue distribution of ENTV-1 provirus in experimentally and naturally infected animals were similar, thereby validating the experimental infection method used in this study and providing further support for the hypothesis that ENTV-1 is the causative agent of ENA in sheep.

## Materials and methods

### Animals, inoculum, and sample collection

The Animal Care Committee at the University of Guelph approved all animal use and related procedures. Five lambs born to dams from a research flock at the University of Guelph with no previous history of ENA were infected at 14 days of age with two mL of ENA inoculum. Two lambs were mock infected with vehicle alone and were housed with the experimentally infected animals. All lambs were examined by a veterinarian and were clinically healthy prior to infection.

The ENA inoculum, which was comprised of cell-free tumor homogenate, was prepared as follows. Equal amounts of tumor tissue (~1 g/tumor) from ten different ENA samples from North America (ENTV-1NA1-10; [3]) were combined and homogenized using a Warring blender. The homogenized tumor cell suspension was diluted in phosphate buffered saline (PBS) (10% w/v) and clarified at 18 000 × *g* for 40 min before concentration by ultracentrifugation in a SW31Ti rotor (Beckman Coulter Canada) at 60 000 × *g* for 2 h at 4°C. The resulting pellet was resuspended in 12 mL of PBS and passed through a 0.45 μm filter.

To ensure efficient delivery of the ENA inoculum to the entire respiratory tract, nebulization was employed. A mask possessing a rubber seal to optimize a tight fit around the nose was fabricated and fitted with an inhaler connector and a one way “T” valve. Conventional 6 mL misty-Neb nebulizer cups (Wilder medical, Kitchener, ON, Canada) were employed to deliver the inoculum. Nebulization was performed using a PM14 compressor (Precision Medical Inc., Northampton, PA, USA).

Peripheral blood mononuclear cells (PBMCs) were collected from infected and control lambs prior to inoculation and every two wpi and stored at −80°C.

Animals were euthanized after the onset of clinical signs or, in the absence of clinical signs, at 45 wpi. At necropsy, the nose was serially cut transversely at ~2 cm intervals and samples of normal conchae and lesions were collected. Tissue samples collected at necropsy included, trachea, lung, submandibular lymph node, liver, spleen, and kidney. These samples were divided in half and stored at −80°C for subsequent isolation of nucleic acids and protein or fixed in 10% neutral buffered formalin for 24 h prior to embedding in paraffin wax and sectioning.

### Computed tomography (CT)

Computed tomography of the head was performed using a GE Bright Speed 16-slice helical CT scanner. The scan parameters were 120 kvp, 200 mA, 1.25 mm slice thickness, and 0.75 pitch. For the CT scan, lambs were anesthetized using routine veterinary methods for this species. Images were interpreted by a board certified veterinary radiologist (HC).

### Histopathology and immunohistochemistry

Formalin-fixed tissues were trimmed, embedded in paraffin, sectioned at 5 μm and processed to obtain haematoxylin-eosin stained sections. The avidin-biotin-peroxidase complex (ABC) method was used on paraffin-embedded tissue sections for immunohistochemical demonstration of ENTV envelope protein expression as described previously [3]. Neoplastic tissue was stained with antibodies to pankeratin (Cell Signaling, clone C11), CK7 (DAKO, clone OVTL 12/30) and vimentin (DAKO, clone V9).

### Heminested PCR (hnPCR)

Genomic DNA was extracted from PBMCs and homogenized tissue samples using the Qiagen DNeasy blood and tissue kit (Qiagen) according to the manufacturer’s instructions. An exogenous ENTV-1 specific hnPCR assay was used to screen for integrated ENTV-1 provirus as described previously [3]. Specificity of the PCR products was confirmed by sequencing. Genomic DNA extracted from the lung tissue of a healthy sheep served as a negative control.

### Buoyant density analysis

Approximately 3 g of tumor tissue was homogenized as described above. Pelleted virus was resuspended in 1 mL of PBS, placed on a linear 20 to 60% (wt/wt) discontinuous sucrose gradient and centrifuged at 100 000 × *g* for 16 h at 4°C in a SW41 rotor (Beckman). 500 μL fractions were collected and their density determined using a refractometer (Fisher Scientific). Reverse transcriptase (RT) activity of each fraction was determined using the EnzChek RT assay kit (Invitrogen) according to manufacturer’s instructions.

### Western blot analysis

Western blot analysis was conducted as described previously [13] using monoclonal antibodies specific for the envelope protein of ovine betaretroviruses (i.e. JSRV, ENTV and enJSRV) [14] and capsid [15] (kindly provided by Dr Hung Fan, University of California, Irvine) proteins.

### Electron microscopy

Buoyant density fractions containing the greatest RT activity were combined, diluted to 30 mL with PBS and placed on a 5 mL 20% sucrose cushion. Virus was pelleted by ultracentrifugation at 18 000 × *g* for 2 h at 4°C and resuspended in 300 μL of HEPES buffer. Virus was then placed on a carbon grid, negatively stained with uranyl acetate and examined using a LEO 912ab transmission electron microscope at the Electron Microscopy Unit, University of Guelph.

### Sequence analysis

Exogenous ENTV-1 specific primers [3] were used to amplify three overlapping fragments, covering the full-length ENTV-1 genome, from a genomic DNA extract of the experimentally induced tumor. The PCR products were sequenced directly and the full genome sequence designated, ENTV-1OVC, was analyzed using the MEGA5 software package.

### Nucleotide sequence accession number

The nucleotide sequence of ENTV-1OVC was deposited in GenBank. Accession number: GenBank:KC189895.

## Results

### Tumor induction in a lamb after inoculation with ENA homogenate

**Figure 1:**
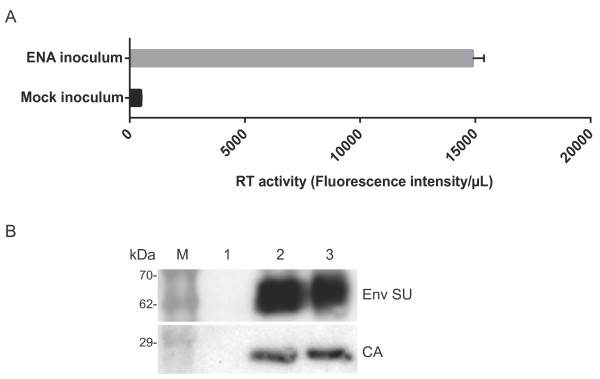
**Reverse transcriptase activity assay and immunoblot analysis of ENA inoculum.** The reverse transcriptase activity of the ENA inoculum was quantified using a fluorogenic based assay **(A)** and compared to a mock inoculum comprised of concentrated filtered supernatant from a sheep skin fibroblast cell line. **(B)** Immunoblot analysis of cell lysate from normal sheep nasal tissue (lane 1), ENA inoculum (lane 2) and cell lysate from a naturally occurring ENA tumor (lane 3) with envelope surface subunit (Env SU) and capsid (CA) specific antibodies. M indicates molecular weight marker.

**Figure 2:**
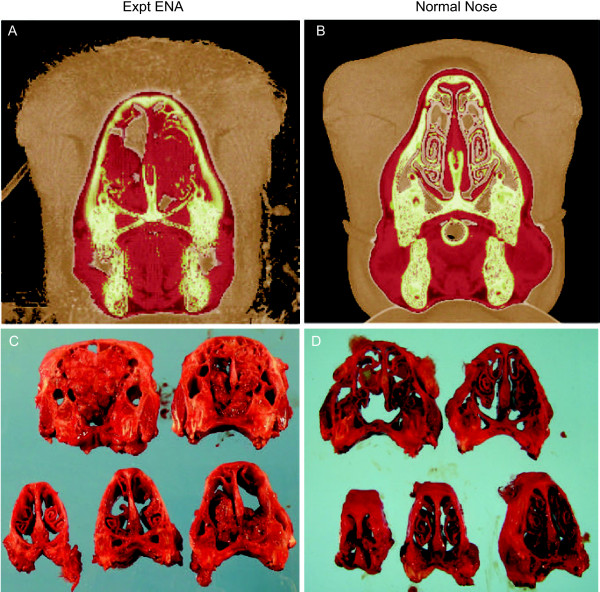
**Computed tomography and gross pathological analysis of sheep experimentally infected with ENA inoculum.** Three dimensional reconstructed computed tomography scan of sheep experimentally infected with ENA inoculum with **(A)** and without **(B)** nasal tumors. Serial sections of the nasal cavity of sheep experimentally infected with ENA inoculum with **(C)** and without **(D)** nasal tumors.

In this study, we tested the hypothesis that ENA could be induced in 14-day-old lambs using a cell free preparation of tumor homogenate derived from naturally occurring cases of ENA in sheep. ENTV-1 was detected in the ENA inoculum by RT activity (Figure 1A) and by immunoblot analysis with antibodies specific for ovine betaretroviral envelope and capsid proteins (Figure 1B). These results confirmed the presence of ENTV-1 antigen and reverse transcriptase activity in the inoculum. ENA inoculum was administered to five 14-day-old lambs (two mL each) using a nebulizer. Clinical signs were observed in one of the five infected lambs at 12 wpi and included persistent nasal discharge, stridor, nostril flaring, head shaking, sneezing and open mouth breathing (Additional file 1). The lamb was euthanized and a post mortem computed tomography (CT) scan was performed (Figure 2A). This showed a bilateral soft tissue density mass with poorly defined margins and a few pinpoint areas of mineralization. The mass was associated with adjacent sinusitis and fluid accumulation within the air spaces and sinuses. The mass was causing destruction of the bony cartilages of the nasal turbinates and occupied approximately 50% of the overall nasal cavity, and approximately 95% of the nasal cavity at the site where it was largest (Figure 2A).

Postmortem findings confirmed the results of the CT scan. The nose of the lamb was sectioned rostral to caudal, exposing a mass within both nasal cavities (Figure 2C). The tumor was approximately five cm in diameter and had a heterogeneous texture and colour, ranging from hard and pink to soft and red. It had several distinct nodules protruding from the surface. The lungs were palpated and inspected for tumors but no lesions were detected. The experiment was terminated at 45 weeks post-infection at which point the remaining four animals were anaesthetized and subjected to CT scan followed by euthanasia and necropsy. No signs of nasal tumor induction were observed in any of the four asymptomatic sheep either by CT (Figure 2B) or necropsy (Figure 2D).

### Histopathological identification of two tumor morphologies in Expt ENA

**Figure 3:**
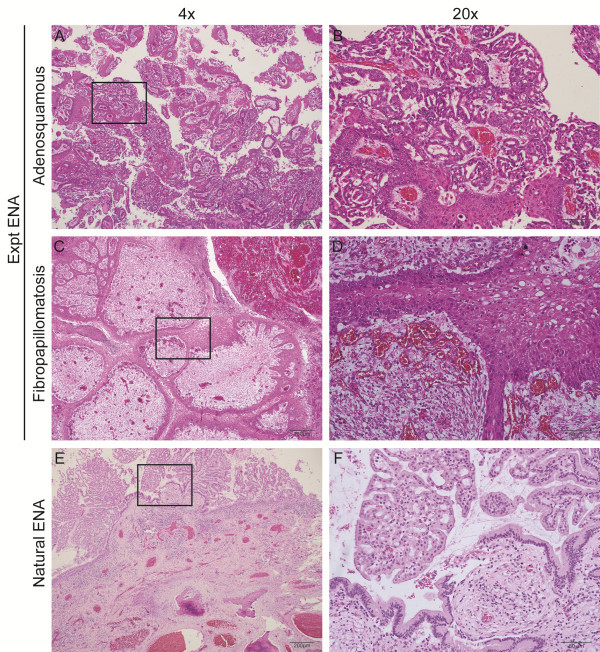
**Histopathology of nasal tumors from sheep experimentally infected with ENA inoculum and naturally acquired ENA.** Representative images of hematoxylin and eosin stained nasal tumors from sheep experimentally infected with ENA inoculum **(A-D)** and naturally acquired ENA **(E, F)**. Representative images showing the adenosquamous **(A, B)** and fibropapilloma **(C, D)** component of the experimentally induced nasal tumor.

Several different regions of the experimentally induced ENA (Expt ENA) tumor were sectioned and separate adenosquamous and fibropapillomatosis components were found. The adenosquamous region (Figure 3A,B) had an outer layer of normally differentiating stratified squamous and keratinising epithelium which connected with underlying glandular epithelial cells that made up most of this component. The neoplastic glandular cells had twofold variation in nuclear size and five mitoses in 10 high-power fields.

The second region had a papillary appearance (Figure 3C,D) with an outer layer of normally differentiating, stratified squamous epithelium with long projections that extend into the underlying abundant fibrous tissue that made up most of this mass.

Tumor tissue from a case of Natural ENA (Figure 3E,F) had similar features to the adenosquamous region of Expt ENA, but lacked features characteristic of the fibropapillomatosis region.

### Immunohistochemical detection of ENTV envelope protein and CK7 in the adenosquamous region of Expt ENA

**Figure 4:**
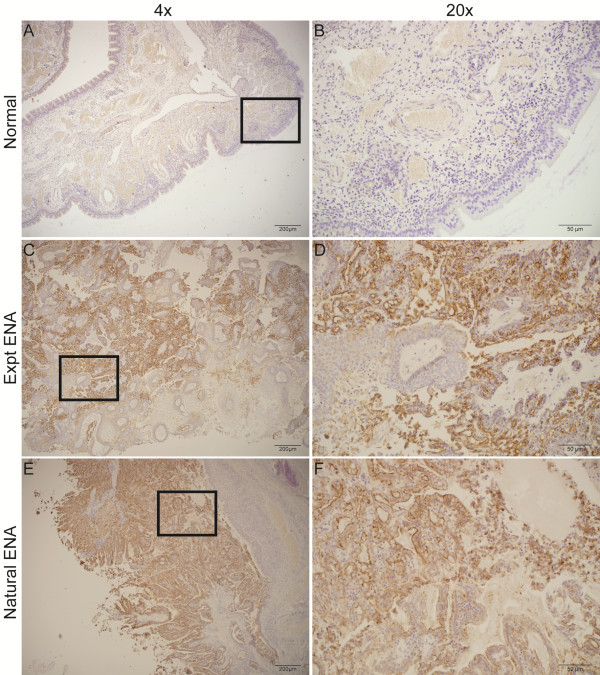
**Immunohistochemical staining for ENTV-1 envelope protein expression in nasal tumors from sheep experimentally infected with ENA inoculum and naturally acquired ENA.** Representative images showing a lack of envelope protein expression in normal sheep nasal epithelium **(A, B)** but robust expression on the apical surface of all cells within the adenosquamous portion of the experimentally induced **(C, D)** and naturally acquired **(E, F)** ENA.

Immunohistochemical staining with a monoclonal antibody specific for the ENTV Env protein of ovine betaretroviruses [14] revealed a complete lack of staining of the normal respiratory mucosa (Figure 4A and B) but intense apical staining of epithelial cells comprising the Expt ENA tumor (Figure 4C and D). Within the tumor, there was strong surface and cytoplasmic staining of all epithelial cells displaying a glandular phenotype, but not of epithelial cells with a stratified squamous differentiation pattern. No specific staining of any of the stratified squamous epithelial cells or of the stroma of the fibropapillomatosis regions was observed. Staining of tumor tissue from a case of Natural ENA showed a very similar pattern (Figure 4E and F).

**Figure 5:**
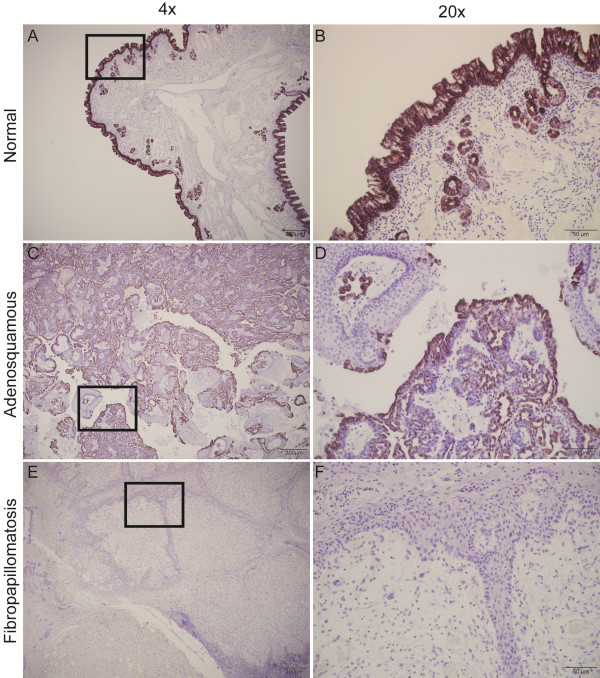
**Immunohistochemical staining for cytokeratin 7 (CK7) in experimentally induced nasal tumors and normal nasal mucosa.** Representative images showing low molecular weight CK7 staining in normal sheep nasal mucosa **(A, B)** as well as in the adenosquamous portion of the experimentally induced nasal tumor **(C, D)** and absence of staining in the fibropapilloma portion of the experimentally induced nasal tumor **(E, F)**.

Staining with an anti-cytokeratin 7 antibody was strongly positive for all epithelial cells of the normal nasal mucosa including glands and luminal epithelial cells (Figure 5A and B). All cells with a glandular phenotype in the adenosquamous portion of the tumor had strong cytoplasmic staining (Figure 5C and D), but none of the cells in the fibropapillomatosis region had positive staining (Figure 5E and F). Since CK7 and envelope expression were observed in the adenosquamous region of the tumor but were lacking in the faibropapillomatosis region this suggests that the adenosquamous region is derived from the tubuloglandular epithelial cells of the nose but that the fibropapillomatosis region is not. The fibropapillomatosis region is likely derived from stromal cell expansion induced to support the growth of the envelope positive adenosquamous tumor region. Immunohistochemical staining for high molecular weight cytokeratin was absent from both the adenosquamous and the fibropapillomatosis regions of the tumor (data not shown).

### Tissue distribution of ENTV-1 proviral DNA

**Figure 6:**
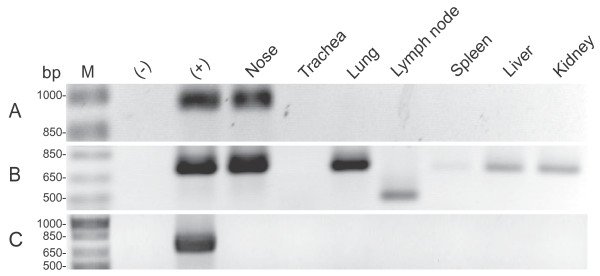
**Tissue distribution of ENTV-1 provirus in sheep experimentally infected with ENA inoculum.** PCR **(A)** and hemi-nested PCR **(B** and **C)** amplification of exogenous ENTV-1 proviral DNA from genomic DNA extracted from various tissues of sheep experimentally infected with ENA inoculum. Distribution of ENTV-1 provirus in an experimentally infected sheep that developed a nasal tumor **(A** and **B)** and in a sheep that did not **(C)**. Genomic DNA isolated from healthy sheep lung (−) and naturally acquired ENA (+) were used as a negative and positive controls, respectively. M indicates molecular weight marker.

Genomic DNA was extracted from the nose, trachea, lung, mandibular lymph node, spleen, liver and kidney of experimentally infected sheep and analyzed for the presence of ENTV-1 proviral DNA using ENTV-1 specific hemi-nested PCR primers [3]. All samples from mock infected sheep were negative for virus (data not shown). ENTV-1 proviral DNA was detected in the nose of the sheep with experimentally induced ENA in the first round of PCR (Figure 6A). In a second round of hemi-nested PCR, proviral DNA was detected in the lung, spleen, kidney and liver, but not in the lymph node or the trachea of the sheep with the experimentally induced ENA (Figure 6B). Interestingly, when DNA extracted from the PBMCs of the experimentally infected sheep that developed a nasal tumor were analyzed using the hnPCR assay, ENTV-1 provirus could not be detected at any time, including two weeks after infection and two weeks prior to euthanasia (data not shown). For the experimentally infected sheep that did not develop ENA, ENTV-1 provirus was absent from all tissues examined (Figure 6C).

Tissues positive for ENTV-1 provirus were examined for viral protein expression by western blot and immunohistochemical staining using an Env-specific monoclonal antibody. Using these methods, ENTV Env protein could only be detected in the nasal tumor tissue of the sheep with ENA (data not shown).

### Ovine betaretroviral virions detected in Expt ENA homogenate by buoyant density analysis and electron microscopy

**Figure 7:**
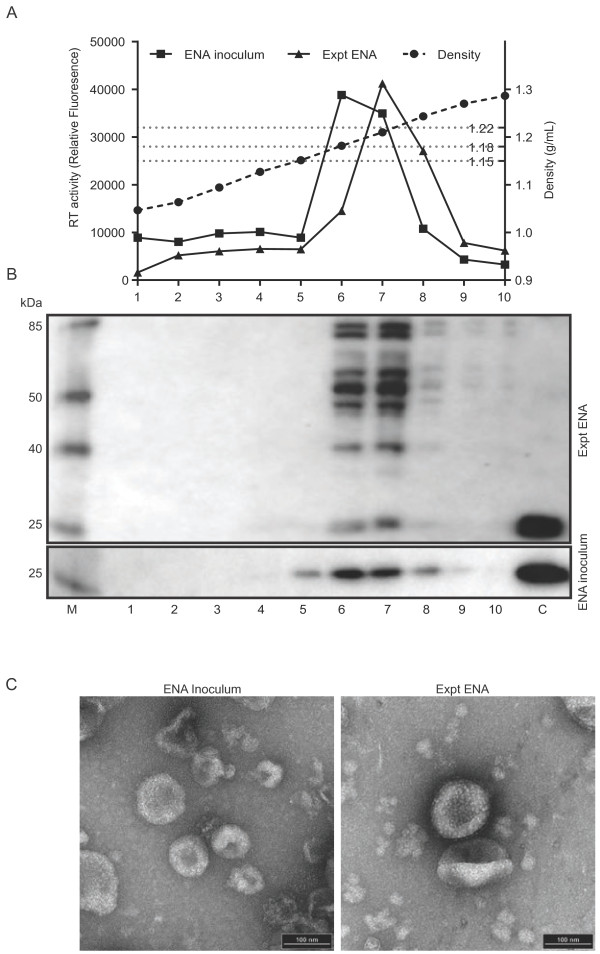
**Buoyant-density and transmission electron micrograph analysis of ENA tumor homogenate. (A)** 20 to 80% (wt/wt) sucrose gradient isopycnic centrifugation of the original ENA inoculum and a preparation of cell free tumor homogenate from the experimentally induced nasal tumor. The RT activity (solid lines) and density (dashed line) of each of the ten fractions is shown. **(B)** Immunoblot analysis for ENTV-1 capsid protein in fractions one through ten. ENA inoculum was loaded as a positive control for capsid (lane C). **(C)** Transmission electron micrograph analysis of fractions six and seven of the ENA inoculum (left) and cell free tumor homogenate from the experimentally induced nasal tumor (Expt ENA; right) showing virus particles with typical betaretrovirus morphology.

Virus purified from the ENA inoculum and the Expt ENA tumor were subjected to buoyant density analysis on a discontinuous 20 to 60% sucrose density gradient. A total of 10 fractions of 500 μL were collected and analyzed for reverse transcriptase activity (Figure 7A), density (Figure 7A), and capsid protein content (Figure 7B). A peak of RT activity was observed in the ENA inoculum in fraction 6, which corresponded to a density of approximately 1.18 g/mL (Figure 7A). The adjacent fraction 7 had a density of 1.22 g/mL and also contained RT activity. By western blot, a 27 kDa protein corresponding to the capsid protein was detected in both fractions 6 and 7 (Figure 7B). Capsid protein was also detected in fraction 5 and 8. The Expt ENA sample showed an increase in RT activity in fraction 6 with a density of 1.18 g/mL; however the majority of RT activity in this sample was found in fraction 7, which corresponded to a density of 1.22 g/mL (Figure 7A). Immunoblot analysis of Expt ENA fractions 6 to 8 revealed the presence of multiple capsid protein products of approximately 85, 78, 62, 57, 52, 40 and 27 kDa (Figure 7B).

On electron micrographic analysis, the fractions from the buoyant density gradient with the highest RT activity (fraction 6 and 7) had virus particles of similar size and morphology in both the ENA inoculum (Figure 7C, left) and Expt ENA (Figure 7C, right) samples. Particles were spherical with a diameter of 80 to 120 nm. Located eccentrically was a spherical electron dense core. The particle surface was rough in appearance with the smooth membrane interrupted at even intervals by structured projections with an apparent trimeric symmetry. There was an abundance of cellular debris on the electron micrograph. No other viral particles were seen.

### ENTV-1 sequences in the experimentally induced tumor are derived from ENTV-1NA9

**Figure 8:**
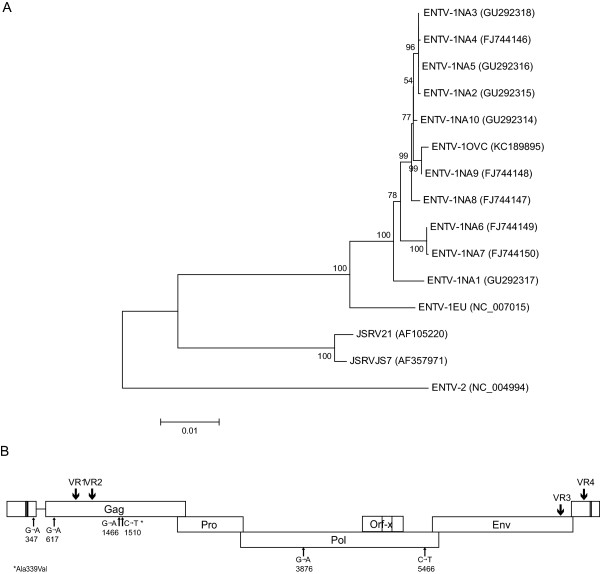
**Phylogenetic analysis of ENTV-1OVC. (A)** Phylogenetic relationship analysis of the full-length ENTV-1 genome sequence from the experimentally induced nasal tumor (ENTV-1OVC) and all other full-length ENTV-1 genome sequences available on GenBank as well as genome sequences from the genetically related ENTV-2 and JSRV. Accession numbers are shown in brackets. The percentage of replicate trees in which the associated taxa clustered together in the bootstrap test (1000 replicates) is shown next to the branches. Phylogenetic analyses were conducted using MEGA5 and all positions containing gaps and missing data were eliminated from the dataset. **(B)** A schematic showing the location of nucleotide differences between ENTV-1OVC and the closely related ENTV-1NA9. Small arrows indicated nucleotide changes and large arrows demarcate regions of variability previously identified.

Overlapping fragments comprising the complete ENTV-1 provirus were amplified from the genomic DNA of Expt ENA tumor tissue and the full-length genome, designated ENTV-1OVC, sequenced. Tissues were taken from separate distinct areas of the nasal tumor and combined for DNA extraction and subsequent amplification. PCR products were sequenced in both directions and as reported previously [3], no variation or evidence of quasispecies was detected. Phylogenetic analysis showed ENTV-1OVC shared a node with ENTV-1NA9 and was thus most closely related to ENTV-1NA9 (Figure 8A). The ClustalW alignment of ENTV-1OVC differed at every nucleotide position in which ENTV-1NA9 differed from the consensus sequence of all ENTV-1 genome sequences available on GenBank. Wherever ENTV-1NA9 and ENTV-1OVC diverged from the consensus sequence, they shared 100% nucleotide identity (data not shown). ENTV-1OVC differed from ENTV-1NA9 at six nucleotide positions and these differences were not found in any ovine betaretrovirus sequences, exogenous or endogenous, currently available on GenBank. The six nucleotide differences were distributed across the genome (Figure 8B) with one occurring in the U5, three in the *gag* gene and two in the *pol* gene. None of the substitutions were located in the four hypervariable regions previously identified in the ENTV-1 genome [3]. All of the nucleotide substitutions represented transition mutations. Only one of the nucleotide differences resulted in a non-synonymous mutation, causing an alanine to valine mutation at amino acid 335 of the *gag* gene.

## Discussion

Although ENA is known to be a transmissible tumor in goats [10], studies on the transmissibility of ENA in sheep are lacking. While a longstanding correlation between ENTV-1 and ENA suggests that this ovine betaretrovirus is the causative agent of the nasal tumor [5,7], Koch’s postulates had not been fulfilled. A nasal tumor with components identical to ENA was induced in an inoculated lamb, thus we provide convincing evidence that ENA is caused by ENTV-1. We used a cell-free ENA tumor homogenate to induce nasal tumors in healthy lambs and found ENTV-1 viral antigens, proviral DNA, and virus particles in the resultant tumor in one of five lambs.

The time to lethal tumor development in our experimental infection trial was surprisingly short. One of five inoculated animals developed clinical signs of ENA at 12 wpi. Normally the estimated time for the development of naturally acquired ENA ranges from one to three years or more. Moreover, in a similar study conducted in goats, the tumor latency was between 12 to 18 months [10].

Nebulization was chosen as the method for virus administration to efficiently distribute the inoculum throughout the upper and lower respiratory tract. ENTV-1 proviral DNA was detected in the nose, lung, spleen, liver and kidney of the animal with experimentally induced ENA suggesting a disseminated distribution. Although ENTV-1 provirus was detected in regions of the respiratory tract other than the nose, this did not correlate with viral protein expression. Our inability to detect protein expression in any of the tissues that were PCR positive for the virus, other than the nose, suggests that either infection of these tissues is non-productive or that infection events in these tissues are so rare that only the use of extremely sensitive methods such as hnPCR are able to detect them. It is possible that the presence of proviral DNA in the spleen, kidney and liver could be due to the migration of virus infected macrophages or dendritic cells to these tissues [16]. At no point during the infection trial was ENTV-1 proviral DNA detected in PBMCs. Ortin et al. were also unable to detect ENTV-1 in the PBMCs of ENA affected animals [8]. In contrast, JSRV is widely distributed both in T and B lymphocytes and mononuclear phagocytes of OPA affected animals [16,17]. Pseudotyping and receptor binding experiments have shown that ENTV-1 and JSRV envelope proteins both bind to and utilize the same receptor, hyaluronidase 2 (Hyal2), for entry [18-21], but the requirements for entry of these viruses have not been evaluated in the context of the native virion. The entry requirements for ENTV-1 and JSRV envelope pseudotyped MLV particles differ slightly as ENTV-1 envelope requires a lower pH than JSRV envelope to mediate entry in this context [22]. It is possible that ENTV-1 requires an additional cofactor for entry that JSRV does not and that this cofactor is lacking in immune cells.

The tissue distribution pattern of ENTV-1 in the experimentally infected sheep with ENA was similar to that reported for naturally acquired ENA [8]. This demonstrates that the infection method used in this study, while likely more efficient, closely recapitulates what is seen during natural transmission of the virus.

Tumor latency was much shorter in our experimentally infected sheep than has been reported in natural cases. This is likely a consequence of the amount of virus in the inoculum and the young age at which the lambs were infected, as a similar reduction in incubation period was observed for newborn lambs experimentally infected with JSRV [23]. Simple retroviruses, such as ENTV-1, require active cell division to translocate their genomes into the nucleus where they integrate in order to complete their replication cycle [24]. Fourteen day old lambs were used in this study since cells in the respiratory tract would be expected to have a higher rate of cell division than adult sheep thereby maximizing infection efficiency. Additionally, the expression level of ovine hyaluronidase 2 (Hyal2), the receptor for ENTV-1, is high in the fetus and then markedly declines in the neonate [25].

The disease incidence was relatively low in our experimental infection trial as only one of five infected animals developed a nasal tumor and for the remaining four animals, ENTV-1 provirus was undetectable by hnPCR. Since our analysis confirmed the presence of ENTV-1 antigens, RT enzymatic activity, and intact virions in the ENA inoculum, it is unlikely that the absence of ENTV-1 proviral DNA in the four asymptomatic sheep was due to a lack of infectious virions in the inoculum. Possible explanations for the low tumor incidence include immune-mediated clearance of virus infected cells and differences in genetic susceptibility to the virus. Indeed, allelic and expression variability of restriction factors and infection co-factors have been shown to influence the replicative fitness of ovine retroviruses [26-30]. As well, certain endogenous ovine betaretrovirus elements restrict JSRV release [31] and have been shown to have variable expression in respiratory tissues [32]. Whether these endogenous elements have similar inhibitory effects on ENTV-1 is currently unknown.

Currently, the immunological response to ENTV-1 infection is poorly understood. ENA affected animals do not appear to develop circulating antibodies towards ENTV-1 [33]. This has been attributed to the immune tolerance induced by expression of endogenous ovine betaretrovirus transcripts in the thymus and peripheral immune organs during ontogeny [34]. Similar studies pertaining to JSRV and OPA have had limited success in detecting a humoral immune response [33]. In sheep experimentally co-infected with JSRV and maedi-visna virus, CD3(+) T cell and JSRV specific antibody responses were detected and these correlated with the spontaneous regression of JSRV-induced lung tumors [35]. A cell-mediated response to ENTV-1 infection has yet to be demonstrated and we did not see evidence of nasal inflammation or tumor related leukocytes in our study. It is possible that the apparent lack of virus specific immune response in ENA affected sheep contributes to tumorigenesis in these animals while sheep that are able to mount a robust immune response against ENTV-1 are capable of clearing virus infected cells before the onset of tumorigenesis.

There was no evidence of quasispecies in the proviral sequence extracted from the Expt ENA tumor (ENTV-1OVC), even though several different areas of the tumor were sampled and PCR products sequenced directly. We previously demonstrated that ENTV-1 genome sequences amplified from North American ENA samples are surprisingly stable [3]. Since the ENA inoculum used in the experimental infection was generated from a combination of ten different North American ENA tumor samples, we were interested to determine which genome sequences would predominate in Expt ENA and the extent of genetic variation. The lack of variation in the ENTV-1OVC sequence suggests that the tumor likely originated from a single integration event. Phylogenetic analysis shows that ENTV-1OVC is most closely related to ENTV-1NA9 and inspection of the ClustalW alignment shows that ENTV-1OVC differs from ENTV-1NA9 at only six nucleotides positions. Recombination was ruled out as a factor for introducing these nucleotide changes because the nucleotide differences seen in ENTV-1OVC relative to ENTV-1NA9 are not shared by any of the other ten sequences represented in the virus inoculum. All six of the nucleotide changes represent transition mutations, which most likely occurred due to base mispairing during reverse transcription of the genome. It is well documented that retroviral reverse transcriptases have low fidelity [36,37], thus it is likely that the RT of ENTV-1 is similarly error prone. Alternatively, the nucleotide differences observed in ENTV-1OVC existed previously in the ENTV-1NA9 tumor and this area of the tumor was not sampled during sequencing. Only one of the ENTV-1OVC nucleotide substitutions corresponded to a nonsynonymous change in the amino acid sequence at residue 335 in the gag polyprotein. It is unlikely that this mutation would affect the structure or function of the resulting protein since the physiochemical properties of valine and alanine are conserved; therefore, it is not likely to confer any advantage in terms of pathogenesis or viral replicative fitness.

Buoyant density analysis of the ENA inoculum fractions showed only one band in the capsid immunoblot at 27 kDa, representing fully processed capsid. Conversely, buoyant density analysis of Expt ENA showed multiple bands on the capsid immunoblot, the size of which correspond well with the molecular weight of the various forms expected of unprocessed gag and gag-pro polyprotein as well as partially processed gag products that contain the capsid subunit [38]. Therefore, it appears that mature as well as immature virions were liberated from the Expt ENA tumor whereas only mature virions were found in the ENA inoculum. The peak RT activity of Expt ENA occurred at a density of 1.22 g/mL whereas the RT activity of ENA inoculum peaked at 1.18 g/mL, the buoyant density expected for a retrovirus. Studies of closely related oncoretroviruses have noted that naked viral cores have a buoyant density of approximately 1.22 g/mL, which is distinct from the buoyant density of enveloped virions (1.15 to 1.18 g/mL) [39]. The immature nature of the virions and the increased buoyant density (1.22 versus 1.18 g/mL) indicate that although a proportion of the Expt ENA virions were enveloped and processed, the majority of virus in the tumor was comprised of naked immature cores. It is possible that the mechanical forces of homogenization sheared cells causing the release of assembled but immature virus particles lacking an envelope membrane. This phenomenon was not observed in the Natural ENA because the fast growth of Expt ENA and relative disorganized structure likely impaired virion release and caused an accumulation of unenveloped virion cores in the cytoplasm of tumor cells. Electron microscopic analysis supports this conclusion as mature enveloped virions were clearly visible in the ENA inoculum, but relatively rare in the virus isolated from in the Expt ENA tumor sample.

Taken together, the fact that the experimentally infected sheep that developed ENA was inoculated with filterable homogenate from ENTV-1 antigen and genome sequence positive tumors strongly suggests that the nasal tumor observed in this animal was caused by ENTV-1, thereby further implicating ENTV-1 as the etiologic agent of transmissible nasal tumors in sheep.

## Competing interests

The authors declare that they have no competing interests.

## Authors’ contributions

SRW and SKW conceived and designed the experiments. PIM participated in the design of the study. DLY, ADM, PIM, and SKW, assisted with the experimental infections and animal handling. SRW, DLY, NMLP and SKW collected and processed the samples. NLMP conducted the immunohistochemical staining. SRW carried out all other experiments. HJC performed and interpreted the CT scans. RAF examined and interpreted the histologic sections. SRW drafted the manuscript and SKW and RAF helped edit the manuscript. All authors read and approved the final manuscript.

## Supplementary Material

Additional file 1 **Clinical signs of lamb with experimentally induced ENA.** This file contains a short movie demonstrating an experimentally infected sheep showing clinical signs of ENA. Click here for file
